# D-dimer and fibrinogen indicate ischemic risk in patients with atrial fibrillation after percutaneous coronary intervention

**DOI:** 10.1186/s12959-024-00610-x

**Published:** 2024-05-21

**Authors:** Diona Gjermeni, Viktoria Anfang, Sofia Szabó, Hannah Vetter, Ana C. Venhoff, Stefan Leggewie, David Hesselbarth, Dietmar Trenk, Martin Buechsel, Dirk Westermann, Christoph B. Olivier

**Affiliations:** 1 Department of Cardiology and Angiology, University Heart Center Freiburg-Bad Krozingen, Faculty of Medicine, University of Freiburg, Freiburg, Germany; 2 Department of Rheumatology and Clinical Immunology, Medical Center, Faculty of Medicine, University of Freiburg, Freiburg, Germany; 3 Institute for Clinical Chemistry and Laboratory Medicine, Medical Center, Faculty of Medicine, University of Freiburg, Freiburg, Germany

**Keywords:** Atrial fibrillation, Percutaneous coronary intervention, Coagulation markers, Antiphospholipid antibodies, d-dimer, Fibrinogen, Prothrombin fragments 1 and 2

## Abstract

**Background:**

This study aimed to evaluate the association of antiphospholipid antibodies (aPL) and conventional markers of coagulation with ischemic and bleeding risk in patients with atrial fibrillation (AF) undergoing percutaneous coronary intervention (PCI).

**Methods:**

In this prospective two-center observational cohort study, patients with AF and an indication for oral anticoagulation (OAC) were enrolled after PCI. Blood was drawn on day 1–3 after PCI. Dilute Russell’s viper venom time was used to determine lupus anticoagulant (LA) in OAC-free plasma. Anti-cardiolipin (aCL) IgG, IgM, and anti-β2-Glycoprotein 1 (aβ2GP1) IgG were analyzed by enzyme-linked immunosorbent assay (ELISA). Fibrinogen (FIB), d-dimer, and prothrombin fragment 1 and 2 (PF 1 + 2) were measured in citrated plasma. The primary ischemic outcome was time to major adverse cardiovascular events (MACE; death, myocardial infarction, or stroke) assessed at 6 months. Bleeding was defined according to International Society on Thrombosis and Haemostasis.

**Results:**

158 patients were enrolled between May 2020 and May 2021 on day 1–3 after PCI. The median age was 78 years (interquartile range [IQR] 72–82), 111 (70%) were male, and 39 (25%) presented with acute coronary syndrome. D-dimer was elevated in 74 (47%) patients, FIB was increased in 40 (25%) and PF1 + 2 in 68 (43%) patients. 32 (20%) patients had ≥ 1 antiphospholipid antibody elevated (aPL; LA: 19 [12%], aCL: 14 [9%], aβ2GP1: 2 [1%]). The presence of aPL was neither significantly associated with MACE (HR 1.46, 95% CI [0.39–5.49], *p* = 0.579), nor bleeding (HR 1.07 [0.30–3.84], *p* = 0.917). Elevated d-dimer was significantly associated with higher risk for MACE (HR 5.06 [1.09–23.41], *p* = 0.038) and major bleeding (HR 7.04 [1.58–31.47], *p* = 0.011). Elevated D-dimer increased the predictive capacity of HAS-BLED for major bleedings (HAS-BLED: AUC 0.71 [0.60–0.83] vs. HAS-BLED + d-dimer: AUC 0.79 [0.70–0.88]; *p* = 0.025). Increased levels of FIB were associated with higher risk for MACE (HR 3.65 [1.11–11.96], *p* = 0.033).

**Conclusion:**

Biomarkers of coagulation might be suitable to assess ischemic and bleeding risk in patients with AF following PCI.

**Supplementary Information:**

The online version contains supplementary material available at 10.1186/s12959-024-00610-x.

## Introduction

Up to 15% of patients with diagnosed atrial fibrillation (AF) undergo percutaneous coronary intervention (PCI) due to coronary artery disease (CAD) [1]. These patients are at high risk for both, ischemic and bleeding events [1]. ESC guidelines recommend the CHA_2_DS_2_-VASC score to assess stroke risk in AF patients and guide antithrombotic therapy [1–3]. The HAS-BLED score is used in clinical practice to evaluate the bleeding risk [1, 2]. Biomarkers and biomarker-based risk scores [4–6] might improve risk prediction of AF patients, particularly in patients who categorized previously as low risk by CHA_2_DS_2_-VASC score [1].

In AF patients with oral anticoagulation (OAC) or in patients undergoing PCI, increased levels of d-dimer [7, 8] or fibrinogen [9–11], respectively associate with cardiovascular events and increase the predictive value of the CHA_2_DS_2_-VASC score [7]. Guidelines recommend direct oral non-vitamin K antagonists (DOAC) in preference to a vitamin K antagonist (VKA) for patients with AF undergoing PCI [1]. In patients with antiphospholipid syndrome (APS), however, the DOAC rivaroxaban increased cardiovascular risk compared with VKA [12]. Antiphospholipid antibodies (aPL) are present in one of ten patients after myocardial infarction (MI) and associate with ischemic risk [13, 14]. Lupus anticoagulant (LA) is a predictor for thrombosis risk [15].

This study aimed to evaluate the association of coagulation markers and aPL with ischemic and bleeding risk in patients with AF and an indication for OAC undergoing PCI. No other studies have evaluated the association of these biomarkers in this specific patient cohort treated with OAC and platelet inhibition.

## Methods

### Study design and population

In this two-center observational cohort study, patients with AF undergoing PCI were enrolled between May 2020 and May 2021. The protocol was approved by the ethics committee of the Albert-Ludwigs-University Freiburg, Germany (registry number 194/20). The study was registered at the German Clinical Trials Register (DRKS00021212). All patients provided written informed consent prior to study participation.

Patients were eligible if they had non-valvular AF with an indication for OAC and received coronary stenting in the last 4 days. All patients received clopidogrel as P2Y12-inhibitor. Recent treatment with prasugrel or ticagrelor (within the last 7 days) as well as use of a GPIIb/IIIa-inhibitor (within the last 24 h) were exclusion criteria. Detailed inclusion and exclusion criteria are listed in Supplemental Table S1. In-hospital treatment and discharge were performed per standard of care.

### Blood sampling, PCI and analyses

Oral anticoagulation was paused before non-urgent PCI. All patients received 70–100 U/kg heparin prior to the intervention. After the initial heparin doses further heparin administrations were adjusted according to the active clotting time (ACT). All patients underwent coronary stenting with at least one drug-eluting stent (DES). All patients were treated with 75 mg clopidogrel after PCI. Antithrombotic therapy was prescribed at the discretion of the interventional cardiologist.

Venous blood was collected using a 21 G butterfly needle (Safety-Multifly®, Sarstedt, Nümbrecht, Germany). Blood samples were drawn on day one to day three after PCI and at least two hours after the morning medication intake. Citrated blood (S-Monovette® Citrat 3.2%, 3 ml, Sarstedt, Nümbrecht, Germany) was obtained to measure aPL (Lupus anticoagulant [LA], anti-cardiolipin [aCL] IgG, IgM, and anti-β2-Gylcoprotein 1 [aβ2GP1] IgG) d-dimer, fibrinogen (FIB) and prothrombin fragment 1 and 2 (PF1 + 2).

Lupus anticoagulant positivity was detected by Dilute Russell’s viper venom time (DRVVT). After centrifugation of citrated blood at 22 °C and 1500 g for 10 min, DOAC-stop® treatment was added to 1 ml plasma according to the manufacturer’s recommendation (Haemochrom Diagnostica GmbH, Essen, Germany) to reduce the impact of DOAC on DRVVT. Plasma samples were frozen in aliquots and stored at -30 °C until measured by LA1 Screening Reagent (LA1) and LA2 Confirmation Reagent (LA2) on Siemens CS-5100 (Siemens, Marburg, Deutschland). A normal ratio of LA1/LA2 between 1.01 and 1.36 was defined as absence of LA. The reference ranges for LA1, LA2 and LA1/LA2 ratio were defined based on internal clinic protocols and according to the suggestion of the manufacturer (Siemens CS-5100).

Serum levels of aCL IgG, IgM and aβ2GP1 IgG were measured by enzyme-linked immunosorbent assay (ELISA) as per internal protocol [16]. Tests were performed according to the manufacturer’s instructions (diagnostic-a GmbH, Ebringen, Germany). The aCL antibody titer was reported as standard IgG, IgM anticardiolipin units (GPL/ ml [norm < 14 U/ml] or MPL/ ml [norm < 10 U/ml] respectively) or standard anti-β2-Gylcoprotein 1 IgG (E/ml [norm < 14 E/ml]) as per internal hospital protocols. Studies suggest that IgG for a2GP1 is a better marker for defining aPL than IgM [17, 18].

D-dimer, FIB, and PF1 + 2 were determined after duplicate centrifugation at 22 °C and 1500 g for 10 min in DOAC-free and platelet poor plasma which were frozen after blood withdraw in aliquots at -30 °C. D-dimer (INNOVANCE®, Siemens, Marburg, Germany) and FIB (Test-Thrombin-Reagent, Siemens, Marburg, Germany) were measured with the Siemens CS-5100 (Siemens, Marburg, Germany).

Elevated d-dimer was defined as > 0.5 mg/fibrinogen equivalent units (FEU) and elevated FIB as > 420 mg/dl. PF1 + 2 (Enzygnost® F1 + 2 monoclonal, Siemens, Marburg, Germany) were measured with the ELx808 Absorbance Reader (Bio-Agilent BioTek, Santa Clara, United States). Levels of PF1 + 2 > 229 pmol/l were defined as elevated.

The reference ranges for PF1 + 2 and FIB were defined according to the manufacturers. The cut-off for elevated d-dimer, was defined based on the cut-off used for the exclusion of venous thrombosis thrombosis at the local hospital.

### Exploratory outcomes and follow-up

The primary outcome was defined as time to major adverse cardiovascular events (MACE; composite of all-cause death, myocardial infarction, or stroke) assessed at 6 months ± 2 weeks [19]. Secondary outcome was time to non-major clinically relevant (NMCR) and major bleeding according to the classification of the International Society of Thrombosis and Haemostasis (ISTH) [20]. Other exploratory outcomes included time to major bleeding [21]. At 6 months ± 2 weeks the participants were contacted by telephone by medical trained personal for outcome assessment. In suspected events, clinical data such as discharge letters, coronary angiography reports, or autopsy reports were obtained, and clinical outcome events were adjudicated by two independent board-certified physician reviewers blinded to the laboratory results. Major discrepancies were resolved by a board-certified cardiologist (CBO) who was also blinded to the laboratory results.

### Statistical considerations

Data are represented as numbers with frequencies for categorical variables and median and IQR for continuous variables. To evaluate if the data was normally distributed, Kolmogorov-Smirnov test was applied. Mann-Whitney U was used to compare medians of two unpaired groups and Chi-squared test was used to compare categorical variables. Pearson’s coefficient was used for correlational analysis. Point biserial correlation between dichotomous and continuous variables was estimated using Pearson’s correlation coefficient as well. Univariate Cox proportional hazard regression or logistic regression was performed to evaluate the association of predictor variables on outcomes. Logistic regression with interaction terms was used to assess the association of combined variables on the outcomes. Optimal cut-offs were analyzed with receiver operating characteristic curves (ROC), 95% confidence intervals for the area under the ROC curve are calculated using the Bamber and Hanley method. ROC curves are compared using nonparametric test of equality. Kaplan-Maier method was used for generating survival curves and differences were analyzed by using log-rank test.

All tests were two-tailed and p-values < 0.05 were considered as statistically significant. Data analyses were performed with Prism 9.3.1 (GraphPad Software, La Jolla, California, USA), SPSS 28.0.0.0 (SPSS Inc., Chicago, Illinois, USA) and Stata 17 (StataCorp LLC, Texas, USA).

## Results

### Patient population and medication

158 patients were enrolled between May 2020 and May 2021 on day one to three after PCI. Basal and procedural characteristics are presented in Table 1. The median age was 78 years (interquartile range [IQR] 72–82), 111 (70%) were male, and 39 (25%) patients presented with acute coronary syndrome (ACS). The median CHA_2_DS_2_-VASC score was 5 (IQR 4–6) and the median HAS-BLED score was 3 (IQR 3–4). 78 patients (49%) had a history of PCI and 31 (20%) had a history of myocardial infarction (Table 1). In this study, 39 (25%) patients underwent PCI because of ACS and the rest were elective PCI. 54 (34%) patients presented with single vessel disease and 25 (16%) patients had left main coronary disease. Patients who received more than one stent had significantly more often a left main disease (13% vs. 3%, *p* = 0.037) and multivessel disease (34% vs. 0%, p = < 0.001) compared with patients treated with only one stent.

**Table 1:** Baseline and procedural characteristics of the population

Baseline characteristics	Total (n = 158)
Male sex	111 (70%)
Age	78 (72–82)
Body mass index [kg/m^2^]	27 (24–31)
CHA_2_DS_2_-VASC score	5 (4–6)
HAS-BLED score	3 (3–4)
Type of atrial fibrillation	
Paroxysmal	93 (59%)
Persistent	31 (20%)
Permanent	34 (21%)
**Blood analysis**	
Creatinine [mg/dl]	1.1 (0.9–1.4)
Leucocytes [10^3^/µl]	7.9 (6.5–9.7)
Thrombocytes [10^3^/µl]	205 (174–267)
Hemoglobin [mg/dl]	12.8 (11.3–14)
**Medical history**	
Transient ischemic attack or stroke	30 (19%)
Periphery artery vascular disease	23 (15%)
Heart failure	45 (29%)
Previous percutaneous intervention	78 (49%)
Intracranial bleeding	5 (3%)
Gastrointestinal bleeding	8 (5%)
GFR (Cockroft-Gault) [ml/min]	70 (60–88)
Arterial hypertension	140 (89%)
Hyperlipidemia	125 (79%)
Diabetes mellitus	55 (35%)
Nicotine abuse	10 (6%)
Coronary artery disease	39 (25%)
**Procedural characteristics**	
Index event	
Elective	119 (76%)
Acute coronary syndrome	39 (25%)
Single vessel disease	54 (34%)
Left main disease	25 (16%)
Implanted stents	
1	79 (50%)
>1	79 (50%)

The values are in number and percentage, n (%) or median (interquartile range) [IQR]. Abbreviations: GFR, Glomerular filtration rate

145 (92%) patients were treated with acetylsalicylic acid (ASA) peri-procedurally. 127 (80%) patients received clopidogrel loading (300 mg: 28 [15%]; 600 mg: 99 [63%]) before PCI. 31 (20%) patients were already on clopidogrel maintenance therapy. Nine Patients (6%) were naive for OAC at the moment of inclusion in the study. OAC was paused in 146 (96%) before PCI and continued from the evening after PCI if no bleedings occurred as per internal protocol. 37 (23%) of the patients received ASA beyond discharge and 155 (98%) of the patients were prescribed an OAC at discharge. 4 (3%) of the patients received VKA at discharge, whereas the rest of the patients were treated with a DOAC. Most of the patients were treated with rivaroxaban 65 (41%) and apixaban 53 (34%). Information regarding the type of DOAC at follow-up is represented in Table S2. At 6 months follow-up, 4 (2.5%) patients deceased and one patient had interrupted P2Y12 inhibitor. 10 (6.3%) patients discontinued OAC because of dialysis or bleeding events.

D-dimer was elevated in 74 (47%) patients (median 0.5 [IQR 0.3-1.0] mg/FEU, fibrinogen equivalent unit). FIB was increased in 40 (25%) and PF1 + 2 in 68 (43%) patients. Overall, median FIB was 362 (IQR 304–423) mg/dl and median PF1 + 2 was 203 (IQR 138–306) pmol/l (Table 2).

**Table 2:** Coagulation markers and antiphospholipid antibodies

Laboratory parameters (Total n = 158)	
**D-dimer, FIB and PF 1 + 2 (n = 155)**	
D-dimer [mg/FEU]	0.5 (0.3-1.0)
Elevated d-dimer > 0.5 [mg/FEU]	74 (47%)
Fibrinogen [mg/dl]	362 (304–423)
Elevated fibrinogen > 420 [mg/dl]	40 (25%)
PF 1 + 2 [pmol/l]	203 (138–306)
Elevated PF 1 + 2 > 229 [pmol/l]	68 (43%)
**Antiphospholipid antibodies – ELISA (n = 157)**	
Anticardiolipin IgG [GPL U/ml]	4 (3–6)
Anticardiolipin IgM [GML U/ ml]	3 (2–4)
Elevated aCL IgG ≥ 14 or IgM ≥ 10	14 (9%)
Anti-beta-2-glycoprotein-1 IgG [E/ml]	2 (2–3)
Elevated aβ2GP1 IgG ≥ 14	2 (1%)
**Lupus anticoagulant – dRVVT (n = 154)**	
LA+	19 (12%)
**Presence of antiphospholipid antibodies**	
1 aPL	30 (19%)
2 aPL	1 (0.6%)
3 aPL	1 (0.6%)

The values are in number and percentage, n (%) or median (interquartile range [IQR]). Abbreviations: FEU, fibrinogen equivalent unit; PF 1 + 2, Prothrombin fragment 1 and 2; LA, lupus anticoagulant; aCL, anticardiolipin; aβ2GP1, Anti-beta-2-glycoprotein-1; dRVVT, dilute Russell’s viper venom time

32 (20%) of the patients had at least one aPL elevated (LA: 19 [12%], aCL: 14 [9%], aβ2GP1: 2 [1%]). One patient (0.6%) had two elevated aPL and one (0.6%) patient had three aPL (Table 2).

### Exploratory outcomes and association of markers with the outcomes

The primary ischemic outcome occurred in 11 (7%) patients and non-major clinically relevant (NMCR) or major bleedings occurred in 23 (15%) of the patients (Table 3).

**Table 3:** Primary ischemic outcome and bleeding outcomes at 6 months ± 2 weeks follow-up

Outcomes	Total n = 158
**Death, myocardial infarction, or stroke**	11 (7%)
Death	7 (4%)
Cardiovascular	2 (1%)
Non-cardiovascular	4 (3%)
Undetermined	1 (1%)
Myocardial infarction	2 (1%)
Stroke	2 (1%)
Hemorrhagic	1 (0.5%)
Undetermined	1 (0.5%)
**NMCR or major bleeding (ISTH)**	23 (15%)
NMCR	9 (6%)
Major	14 (9%)
**Any bleeding**	57 (36%)
**Minor bleeding (ISTH)**	34 (22%)

The values are in number and percentage, n (%). Abbreviations: NMCR, non-major clinically relevant; ISTH, International Society on Thrombosis and Hemostasis

**Fig. 1:**
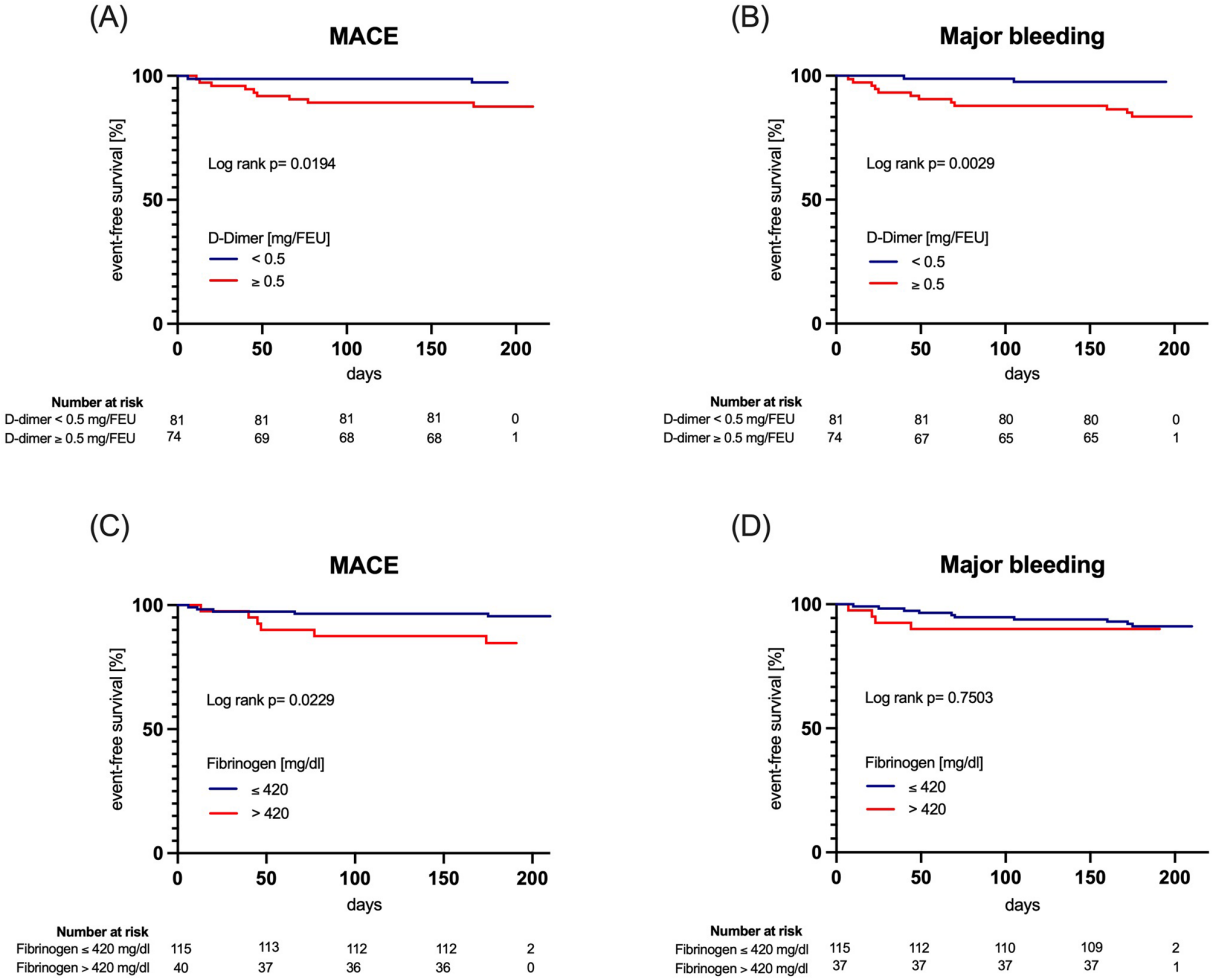
Kaplan-Meier survival curve for the ischemic outcome MACE (**A, C**) and major ISTH bleeding (**B, D**) at 6 months follow up (+/- 14 days) in relation to elevated d-dimer [≥ 0.5 mg/FEU] (**A, B**) or fibrinogen [> 420 mg/dl] (**C, D**), respectively

MACE occurred more frequently within the first 60 days in patients with increased d-dimer and FIB compared with patients with normal d-dimer and FIB (Fig. 1).

**Fig. 2:**
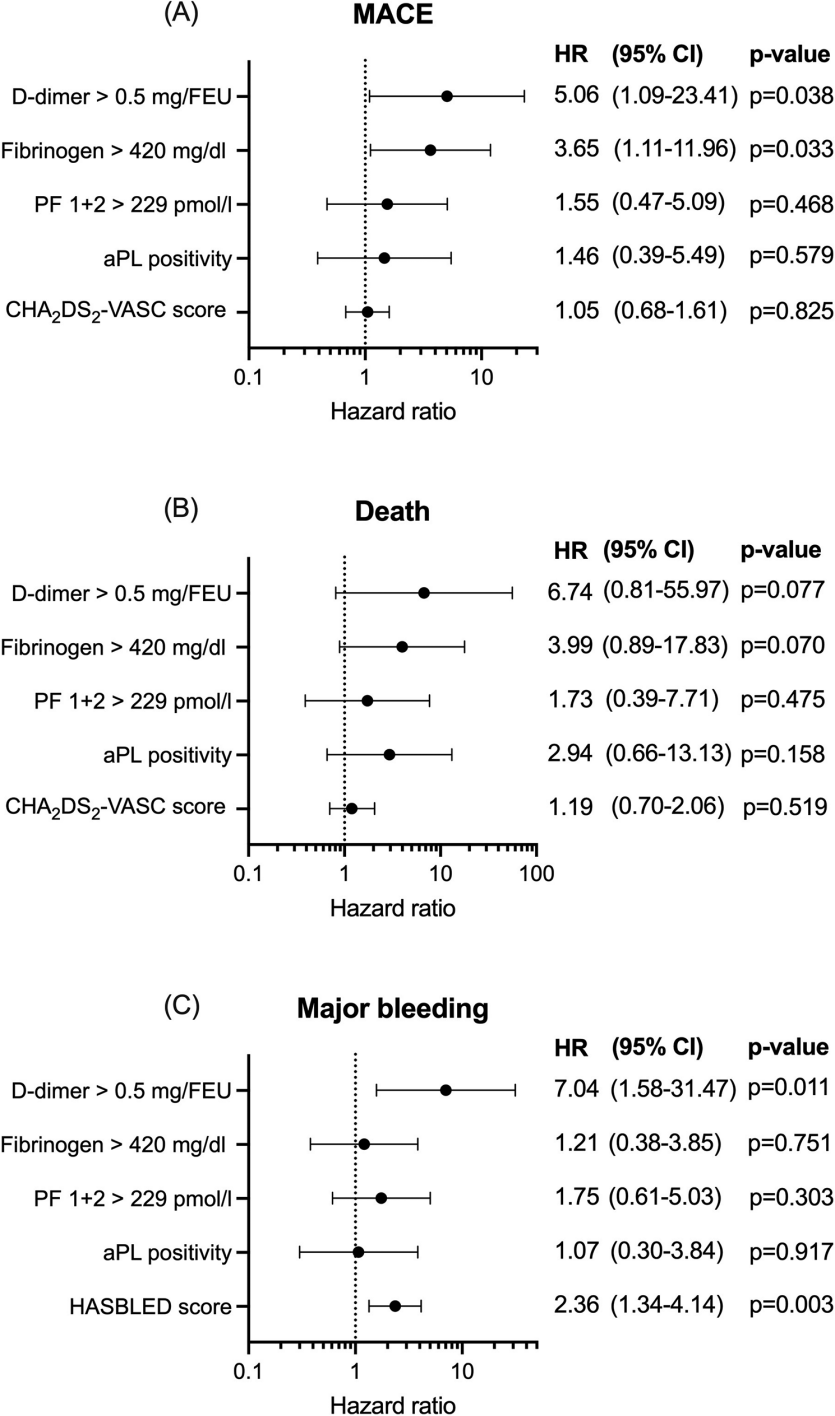
Association of coagulation markers, aPL and risk scores with ischemic and bleeding outcomes. Abbreviations: MACE, major adverse cardiac events; HR, hazard ratio; PF, prothrombin factor; aPL, antiphospholipid antibodies

Increased d-dimer significantly associated with increased risk of MACE (HR 5.06, 95% CI [1.09–23.41], *p* = 0.038) and major bleedings (HR 7.04, 95% CI [1.58–31.47], *p* = 0.011) (Fig. 2). Combining d-dimer with age did not associate with increased risk of MACE (*p* = 0.253) as well as with the secondary bleeding outcome (*p* = 0.651).

D-dimer was significantly higher in patients experiencing MACE compared to patients without an ischemic event (1.1 [IQR 0.7–1.6] vs. 0.5 [IQR 0.3–0.9], *p* = 0.011), (Table S4, supplement). D-dimer was significantly higher in patients with major bleeding event compared with patients without a major bleeding (0.9 [IQR 0.5–1.4] vs. 0.5 [IQR 0.3–0.9], respectively, *p* = 0.011), (Table S5, supplement).

Increased FIB associated with the occurrence of MACE (HR 3.65, 95% CI [1.11–11.96], *p* = 0.033), (Fig. 2). FIB did not associate with major bleedings (Fig. 2).

Elevated d-dimer and elevated FIBC combined were associated with the composite ischemic outcome (OR 5.17, 95% CI [1.44–18.53], *p* = 0.012). The association of these combined variables with the secondary bleeding outcome (OR 1.56, 95% CI [0.52–4.67], *p* = 0.431) and death at 6 months (OR 4.29, 95% CI [0.90-20.52], *p* = 0.068) was not statistically significant. Neither elevated PF 1 + 2, nor the presence of aPL associated with ischemic or bleeding outcome (Fig. 2).

### Association of patient and procedural characteristics with coagulation markers and outcomes

Duration of triple antithrombotic therapy (TAT) correlated with FIB (*r* = 0.161, *p* = 0.046) and time in hours between clopidogrel loading and blood withdrawal correlated moderately with PF1 + 2 (*r* = 0.238, *p* = 0.007), (Table S3, supplement). Correlations between basal characteristics and coagulation factors are shown in supplemental Table S3.

The number of implanted stents (> 1 stent) did not improve the predictive value of d-dimer in predicting MACE (OR 2.56, 95% CI [0.74–8.81], *p* = 0,137). Combining number of implanted stents (> 1 stent) with elevated FIB associated with increased MACE (OR 3.78, 95% CI [1.07–13.34], *p* = 0.039). The combined variables with d-dimer (high variability in OR and high standard error because of the low rate of events, *p* = 0.996) and FIB (OR 4.10, 95% CI [0.25–67.45], *p* = 0.323) did not associate with an increased risk for myocardial infarction.

In the first 30 days after PCI, MACE occurred in 4 patients (2.5%) and major bleeding in 5 (3.2%). No significant association was found between the duration of ASA therapy with short term (≤ 30 days) occurrence of MACE (*p* = 0.657) or major bleeding (*p* = 0.723).

### Coagulation markers and other standard scores

Risk for major bleedings was associated with an increase in HAS-BLED score (HR 2.36, 95% CI [1.34–4.14], *p* = 0.003). There was no association between increased CHA_2_DS_2_-VASC score and ischemic risk (HR 1.05, CI 95% [0.68–1.61], *p* = 0.825) (Fig. 2).

At a cut-off of > 0.67 mg/FEU d-dimer predicted MACE with a moderate test accuracy (AUC = 0.73, 95% CI [0.56–0.90], *p* = 0.013), (Figure S1, supplement). D-dimer at a cut-off > 0.51 mg/FEU predicted major bleedings with an accuracy of (AUC = 0.70, 95% CI [0.59–0.89], *p* = 0.013) and sensitivity of 86% and specificity of 57%. When even more elevated (cut-off 0.87), d-dimer had significant diagnostic power of predicting all-cause mortality (AUC = 0.79, 95% CI [0.64–0.95], *p* = 0.009) with a good sensitivity and low specificity (Figure S1, supplement).

FIB and PF1 + 2 had no statistically significant power to accurately predict any bleeding outcomes (Figure S1, supplement).

HAS-BLED score had a statistically significant moderate ability to predict major bleedings at a cut-off > 3.5 with sensitivity 71% and specificity 63%, (Figure S1, supplement).

CHA_2_DS_2_-VASC score correlated with FIB (*r* = 0.192, *p* = 0.016), whereas HAS-BLED score did not correlate with any of the coagulation markers, (Table S3, supplement).

The predictive value of CHA_2_DS_2_-VASC score for the composite outcome stroke, death and myocardial infarction increased when adding d-dimer > 0.5 mg/FEU to the score (AUC = 0.51, 95% CI [0.33–0.69] vs. AUC = 0.58, 95% CI [0.42–0.75], *p* = 0.015). Furthermore CHA_2_DS_2_-VASC score prediction of death increased by adding d-dimer as well (AUC = 0.57, 95% CI [0.37–0.77] vs. AUC = 0.65, 95% CI [0.50–0.81], *p* = 0.020), (Table S6, supplement).

The ability of HAS-BLED to predict major bleedings increased significantly when adding d-dimer > 0.5 mg/FEU to the score (AUC = 0.71, 95% CI [0.60–0.83] for HAS-BLED vs. AUC = 0.79, 95% CI [0.70–0.88] when adding d-dimer to HAS-BLED, *p* = 0.025), (Table S6, supplement).

## Discussion

The main findings of this study are that (1) increased d-dimer and fibrinogen associated with higher ischemic risk in patients with AF undergoing PCI (2), aPL positivity did not associate with outcomes (3) increased d-dimer associated with higher risk for major bleedings and (4) the ability of HAS-BLED to predict major bleedings increased significantly when adding d-dimer > 0,5 mg/FEU to the score.

### Coagulation markers and association with clinical outcomes

Patients with AF undergoing PCI and an indication for OAC are at high ischemic and bleeding risk a priori, confirmed by a median of CHA_2_DS_2_-VASC score in this study was 5 (IQR 4–6) representative for the high-risk elderly population with AF undergoing PCI.

Coagulation markers such as d-dimer is increased in patients with acute coronary syndrome who undergo stent implantation [22]. 70% of the patients from the ARISTOTLE trial with AF and CHA_2_DS_2_-VASC score between 3 and 5 and treated with a DOAC had increased d-dimer varying from 0.66 to 1.12 mg/FEU [7]. D-dimer also increases with age (> 80 years) in patients with chronic AF [23]. Median d-dimer in the present study was slightly lower (median 0.5 mg/FEU) compared with other studies [7, 19, 23]. D-dimer levels are lower in patients on antithrombotic therapy suggesting d-dimer to indicate fibrin turnover [8, 23]. In this study, 92% of the patients were under triple therapy (ASA, clopidogrel and OAC) during hospitalization due to a recent stent implantation. Increased fibrinogen with values in range 126–696 mg/dl associated with higher risk of coronary artery disease and all-cause mortality [10, 11, 24].

Patients with AF an CHA_2_DS_2_-VASC score > 1 had fibrinogen concentration higher than 323 ± 76.4 mg/dl vs. patients with CHA_2_DS_2_-VASC score < 1 [25]. Similar to other studies, in our patient cohort with high CHA_2_DS_2_-VASC score the mean value of fibrinogen was 362 (304–423) mg/dl and was increased above the reference value in 25% of the patients [10, 11, 25]. D-dimer > 0.3 mg/FEU in patients with AF is associated with increased thromboembolic risk [26, 27]. This study did not identify a significant association of d-dimer with stroke in patients with AF undergoing PCI, but an increased risk of MACE. High d-dimer in patients with AF associated with more occurrence of cardiovascular events [28]. For patients with coronary artery disease high d-dimer at baseline was associated with the risk for death with relative risk (RR) 1.69 and MACE with RR 2.37 [29]. Mortality for patients undergoing PCI due to ACS at 6 months was higher if d-dimer was increased (2.9% mortality for high d-dimer vs. 0.9% when d-dimer was low) [22]. In this study, d-dimer > 0.5 mg/FEU in patients with AF undergoing PCI associated with increased risk of MACE with and there was a trend to associate with increased risk of death. In patients with AF undergoing PCI, d-dimer associated with increased major bleeding risk with HR 7.04 (95% CI [1.58–31.47], *p* = 0.011).

In contrast, one study included patients with COVID who received anticoagulation and demonstrated that d-dimer was lower in patients with major bleeding complications. Nevertheless, most bleedings occurred under a heparin overdose in these patients [30]. In this study, only one of the included patients had COVID and there occurred neither ischemic not bleeding events. Consistent with these findings, a study identified that increased d-dimer in patients with AF associated with 2–3 fold increased risk of major bleeding [7]. The findings of this study were consistent with other studies that considered separate cohorts of either patients with AF, or CAD that suggest an increased risk of cardiovascular events and mortality in patients with increased d-dimer [8, 22, 23, 26–29]. No other studies in the literature have evaluated the association of this pattern of coagulation markers in patients with both AF and PCI.

High levels of FIB (> 420 mg/dl) associated with ischemic outcome. This finding is consistent with other studies. In the Framingham study, the incidence of coronary heart disease was higher in patients with fibrinogen > 312 mg/dl [10]. High fibrinogen associated significantly with risk for CAD and all-cause mortality [11]. Fibrinogen was higher in patients with ST-elevation myocardial infarction (STEMI) compared with patients presenting with CAD [9]. Fibrinogen levels have not previously been described to be a prognostic factor in patients with AF and data regarding association with stroke are discordant [10, 11]. In this study adding d-dimer to fibrinogen associated with a 5-fold increased risk for MACE in patients with AF undergoing PCI at 6 months.

Lower fibrinogen levels are observed in patients with bleeding and fibrinogen is essential to maintain hemostasis [31], in this study, FIB did not associated with bleeding risk.

Other factors such as neutrophil extracellular traps consisting of nucleic acids (DNA and RNA) and proteins have been shown to strongly associate with arterial thrombus formation and stability [32]. We acknowledge that numerous other factors are implicated in the process of arterial thrombosis associating with both ischemic and bleeding risk in cardiologic patients. Further studies are necessary to better assess the association of different biomarkers with clinical outcomes.

### Association between the presence of aPL and clinical outcomes

In patients with APS as well as patients with AF, stroke is the main thromboembolic risk [1, 14]. One study [12] investigated the effectiveness of rivaroxaban compared with warfarin in patients with triple positivity for aPL and history of thrombosis. Rates of ischemic events were higher in the rivaroxaban arm which indicates why testing for aPL might be reasonable in this patient cohort. DOACs which are commonly used in AF patients are not recommend in patients with definite APS and arterial events [33]. Another study demonstrated that in patients with triple positivity for aPL male sex was an independent predictor for thromboembolic events [34]. In this study, positivity for aPL was tested only once whereas Sapporo classification criteria of APS recommend to test on two or more occasions with at least 12 weeks in between [35, 36], which may have led to a possible transient positivity of aPL in this study. This study was observational and the antibodies were only assessed in a setting as exploratory markers. We acknowledge that the measurement of antibodies in the acute thrombotic phase might have altered the results for aPL positivity and this is an important limitation of this study. This study was potentially underpowered to assess an association between the presence of aPL and ischemic and bleeding outcome. Even though, patients with AF, showing positivity for aPL and treated with DOACs might be at a substantial higher risk for thromboembolic and bleeding events [12].

### Coagulation markers and other standard scores

In this study d-dimer achieved a considerable high test accuracy in predicting MACE and death. While d-dimer and HAS-BLED both established a moderate predictive value for major bleedings. Corresponding low predictive values of CHA_2_DS_2_-VASC score for death and MACE in patients with AF and PCI were described by Puurunen et al. [37] with an AUC of 0.57. In the mentioned work HAS-BLED score failed in predicting major bleedings in contrast to this study.

The addition of d-dimer to the well-known risk scores like CHA_2_DS_2_-VASC or HASBLED resulted in an improvement of test accuracy for the prediction of MACE and death by CHA_2_DS_2_-VASC and of major bleeding by HASBLED score, respectively. This is supported by data from Christersson et al. [7] who described an improvement of C-index for predicting death and systemic embolism by adding d-dimer to CHADS_2_/ CHA_2_DS_2_-VASC score and for major bleedings by adding it to the HAS-BLED score.

### Strength and limitations of the study

This is the first study to evaluate the association of this panel of coagulation markers and aPL in patients undergoing PCI with AF and an indication for OAC. The main limitation is the small population size that limits the statistical power of the results. The coagulation parameters were determined as other exploratory markers as part of another study and consequently no sample size calculation was performed, based on data regarding the capacity of coagulation markers to predict ischemic and bleeding risk. No information regarding cancer or autoimmune disorders in patients’ medical history were collected in this study. This study is an observational pilot study and should be regarded as hypothesis generating. Different aPL antibodies were measured only once after PCI and no serial measurements have been performed. Thus, associations were assessed in relation to elevated levels of aPL rather than a diagnosis of APS. Another limitation of the study is that for Lupus anticoagulant only one assay (DRVVT-Test) was performed based on internal clinic protocols. Other biomarkers, such as the presence of neutrophil extracellular traps (NETs), have already been shown to influence the promotion of thrombosis and thrombin generation but were not investigated in this study [32]. Further studies are necessary to investigate the impact of coagulation markers and aPL on ischemic and bleeding risk in patients with PCI and AF.

## Conclusion

Increased d-dimer and FIB associated with ischemic risk in patients with AF undergoing PCI. D-dimer was predictive for mortality in this patient cohort. Increased d-dimer associated with higher major bleeding risk and improved a conventional risk score for bleeding risk assessment. A combined panel of coagulation biomarkers might be suitable to improve identification of patients with AF following PCI at risk for subsequent ischemic and bleeding events. aPL positivity did not significantly associate with ischemic and bleeding risk but the results were inconclusive due to an underpowered study design.

### Electronic supplementary material

Supplementary material

## Data Availability

Not applicable.
