# Supplementary material for: D-dimer and fibrinogen indicate ischemic risk in patients with atrial fibrillation after percutaneous coronary intervention
Source: Thromb J. 2024 May 21;22:42. doi: 10.1186/s12959-024-00610-x (PMC11107060; doi:10.1186/s12959-024-00610-x)

# SUPPLEMENT

## Table S1. Inclusion and exclusion criteria

| **Inclusion criteria** |
| --- |
| - Informed consent - ≥18 years of age or older - Atrial fibrillation with an indication for oral anticoagulation (i.e., CHA2DS2VASC score ≥1 for males, ≥2 for females) - Percutaneous coronary intervention in the first 3 Days |
| **Exclusion criteria** |
| - Contraindication to a DOAC (i.e. apixaban, dabigatran, edoxaban, rivaroxaban) or clopidogrel - History of stent-thrombosis - Uncompliant patient from the point of view of the principal investigator like for example.: malcompliance, very often use of alcohol and drugs or not willing to proceed according to the protocol (patient willing not to be follow-uped) - GPIIb/IIa inhibition in the last 24h - Use of prasugrel or ticagrelor in the last 7 day |

## Table S2. Peri-procedural medication, medication at discharge and at 6 months ± 2 weeks follow-up

| **Medication** | Total  N= 158 | |
| --- | --- | --- |
| **(Peri-)procedural** |  |  |
| ASA |  |  |
| No ASA | 11 | (7%) |
| ASA maintenance therapy | 14 | (9%) |
| ASA Loading | 131 | (83%) |
| ASA beyond discharge only | 2 | (1%) |
| Clopidogrel |  |  |
| Clopidogrel maintenance therapy | 28 | (18%) |
| Clopidogrel loading | 130 | (82%) |
| **At discharge** |  |  |
| ASA | 37 | (23%) |
| ≤ 7 days | 16 | (43%) |
| 8-30 days | 15 | (41%) |
| > 30 days | 6 | (16%) |
| Clopidogrel | 158 | (100%) |
| ≤ 6 months | 4 | (3%) |
| 6 months | 62 | (39%) |
| 9 months | 2 | (1%) |
| 12 months | 90 | (57%) |
| Oral anticoagulation | 155 | (98%) |
| Apixaban | 53 | (34%) |
| Dabigatran | 6 | (4%) |
| Edoxaban | 18 | (11%) |
| Rivaroxaban | 74 | (47%) |
| Vitamin-K-antagonist | 4 | (3%) |
| **At 6 months follow-up (+/- 2 weeks)** |  |  |
| ASA | 7 | (4%) |
| P2Y12-inhibiton | 138 | (87%) |
| Clopidogrel | 136 | (86%) |
| Prasugrel | 2 | (1%) |
| Oral anticoagulation | 148 | (93%) |
| Apixaban | 56 | (35%) |
| Dabigatran | 6 | (4%) |
| Edoxaban | 16 | (10%) |
| Rivaroxaban | 65 | (41%) |
| Vitamin-K-antagonist | 5 | (3%) |
| The values are in number and percentage, n (%). Abbreviation: ASA, acetylsalicylic acid. | | |

## Table S3. Correlation between patient characteristics and coagulation markers

|  | **D-dimer** [mg/FEU] | | **FIB** [pmol/l] | | **PF 1+2** [mg/dl] | |  |  |
| --- | --- | --- | --- | --- | --- | --- | --- | --- |
| Characteristics | r | p-value | r | p-value | r | p-value | |  |
| CHA_2_AS_2_-VASC Score | 0.145 | 0.072 | **0.192** | **0.016** | -0.058 | 0.473 | |  |
| HAS-BLED Score | 0.146 | 0.070 | 0.071 | 0.380 | 0.030 | 0.709 | |  |
| Heart failure | 0.017 | 0.836 | 0.140 | 0.081 | **-0.191** | **0.017** | |  |
| Previous stent | 0.069 | 0.397 | 0.004 | 0.962 | **0.163** | **0.043** | |  |
| Previous MI | 0.132 | 0.102 | 0.056 | 0.488 | **0.184** | **0.022** | |  |
| Previous TIA or stroke | 0.133 | 0.099 | 0.071 | 0.380 | -0.046 | 0.567 | |  |
| History of GI-bleeding | 0.041 | 0.610 | 0.076 | 0.346 | 0.050 | 0.538 | |  |
| Diabetes | 0.012 | 0.879 | 0.056 | 0.487 | -0.082 | 0.312 | |  |
| ACS | 0.075 | 0.352 | 0.100 | 0.216 | -0.051 | 0.531 | |  |
| ASA Loading | 0.034 | 0.677 | 0.052 | 0.523 | -0.016 | 0.839 | |  |
| Duration of TAT | -0.012 | 0.879 | **0.161** | **0.046** | -0.108 | 0.181 | |  |
| Time between Clopidogrel loading and blood withdraw (h) | -0.076 | 0.391 | 0.044 | 0.623 | **0.238** | **0.007** | |  |
| Abbreviations: r, pearson correlation coefficient; FIB, Fibrinogen; PF 1+2, prothrombin fragments 1 and 2; MI, myocardial infarction; TIA, transient ischemic attack; GI, gastrointestinal; ACS, acute coronary syndrome; ASA, acetylsalicylic acid; TAT, triple antithrombotic therapy. | | | | | | | |  |
|  | | | | | | | | |

## Table S4. Coagulation markers and distribution of medians according to the occurrence of MACE (myocardial infarction, death, or stroke) or death

| **Total n= 155** | **MACE**  n= 11 | | **No** **MACE**  n= 144 | | **p-value** |
| --- | --- | --- | --- | --- | --- |
| D-dimer [mg/ FEU] | 1.1 | (0.7-1.6) | 0.5 | (0.3-0.9) | **0.011** |
| Fibrinogen [mg/dl] | 423 | (274-472) | 356 | (304-420) | 0.363 |
| PF 1+2 [pmol/l] | 250 | (123-403) | 199 | (140-303) | 0.417 |
|  |  |  |  |  |  |
|  | **Death**  n= 7 | | **No death**  n= 148 | |  |
| D-dimer [mg/ FEU] | 1.5 | (0.9-1.7) | 0.5 | (0.3-1.0) | **0.007** |
| Fibrinogen [mg/dl] | 444 | (274-472) | 356 | (304-420) | 0.221 |
| PF 1+2 [pmol/l] | 250 | (123-583) | 199 | (140-304) | 0.476 |
| \| The values are represented as median (interquartile range). Abbreviations: PF 1+2, Prothrombin fragments 1 and 2; MACE, major adverse cardiovascular events; \| \| --- \| | | | | | |

## Table S5. Coagulation markers and median distribution according to the occurrence of bleeding outcomes

| **Total n= 155** | **NMCR or major bleeding** (ISTH) n= 23 | | **No NMCR or major bleeding** (ISTH) n= 132 | | **p-value** |
| --- | --- | --- | --- | --- | --- |
| D-dimer [mg/ FEU] | 0.6 | (0.4-1.0) | 0.5 | (0.3-1.0) | 0.251 |
| Fibrinogen [mg/dl] | 334 | (269-413) | 364 | (306-423) | 0.155 |
| PF 1+2 [pmol/l] | 248 | (185-355) | 193 | (129-303) | 0.096 |
|  |  |  |  |  |  |
|  | **NMCR bleeding** (ISTH)  n= 9 | | **No NMCR bleeding** (ISTH)  n= 146 | | **p-value** |
| D-dimer [mg/ FEU] | 0.4 | (0.3-0.5) | 0.5 | (0.3-1.0) | 0.186 |
| Fibrinogen [mg/dl] | 278 | (250-388) | 362 | (305-423) | 0.071 |
| PF 1+2 [pmol/l] | 246 | (162-310) | 202 | (135-307) | 0.731 |
|  |  |  |  |  |  |
|  | **Major bleeding** (ISTH)  n= 14 | | **No major bleeding** (ISTH)  n= 141 | | **p-value** |
| D-dimer [mg/ FEU] | 0.9 | (0.5-1.3) | 0.5 | (0.3-0.9) | **0.011** |
| Fibrinogen [mg/dl] | 340 | (281-446) | 362 | (304-423) | 0.773 |
| PF 1+2 [pmol/l] | 276 | (201-429) | 197 | (130-303) | 0.075 |
|  |  |  |  |  |  |
| The values are median (interquartilrange). Abbreviations: PF 1+2, Prothrombin fragments 1+2; NMCR; Non-major clinically relevant bleeding, ISTH, Internationl Society of Thrombosis and Haemostasis; | | | | | |

**Table S6. Comparison of CHA2DS2-VASC and HAS-BLED score with CHA2DS2-VASC+ and HAS-BLED+ score (including d-dimer > 0.5 mg/FEU) by comparing AUC (95%CI) of receiver operating characteristic curve (ROC) analysis**

|  | CHA_2_DS_2_-VASC | | CHA_2_DS_2_-VASC+ | |  |
| --- | --- | --- | --- | --- | --- |
| Outcome | AUC | 95%CI | AUC | 95%CI | p-value |
| Stroke, death, myocardial infarction | 0.51 | 0.33; 0.69 | 0.58 | 0.42; 0.75 | 0.015 |
| Death | 0.57 | 0.37; 0.77 | 0.65 | 0.50; 0.81 | 0.020 |
| Myocardial infarction | 0.20 | 0.00; 0.41 | 0.33 | 0.09; 0.57 | 0.000 |
| Stroke | 0.62 | 0.01; 1.00 | 0.58 | 0.00; 1.00 | 0.567 |
| Major bleeding | 0.50 | 0.36; 0.64 | 0.59 | 0.45; 0.73 | 0.001 |
|  |  |  |  |  |  |
|  | HAS-BLED | | HAS-BLED+ | |  |
| Outcome | AUC | 95%CI | AUC | 95%CI | p-value |
| Stroke, death, myocardial infarction | 0.61 | 0.43; 0.78 | 0.69 | 0.51; 0.86 | 0.019 |
| Death | 0.75 | 0.62; 0.88 | 0.82 | 0.70; 0.94 | 0.157 |
| Myocardial infarction | 0.26 | 0.00; 0.53 | 0.42 | 0.12; 0.73 | 0.000 |
| Stroke | 0.44 | 0.00; 1.00 | 0.48 | 0.00; 1.00 | 0.644 |
| Major bleeding | 0.71 | 0.60; 0.83 | 0.79 | 0.70; 0.88 | 0.025 |
|  |  |  |  |  |  |
|  |  |  |  |  |  |

## Figure S1. Forrest plot of receiver operating characteristic curve (ROC) analysis of coagulation markers, antiphospholipid antibodies, and risk scores (CHA2DS2-VASC score, HAS-BLED score)


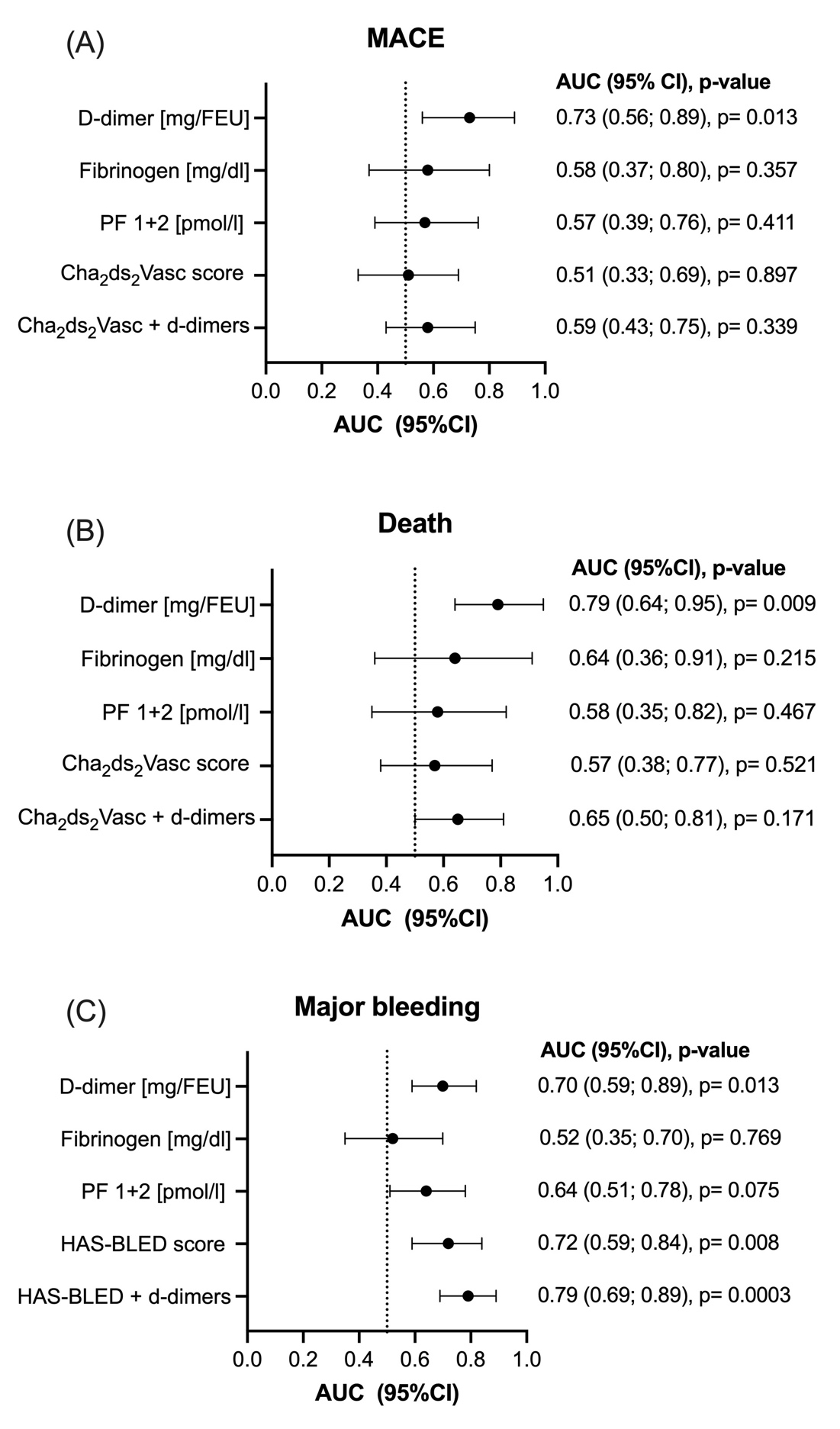

Supplement: Supplementary file 1 — Supplementary material [file 12959_2024_610_MOESM1_ESM.docx]
